# Relapsing De Novo Membranous Nephropathy

**DOI:** 10.1155/2022/6754520

**Published:** 2022-05-02

**Authors:** Ala Ali, Huda Al-Taee, Thaer J. Kadhim

**Affiliations:** ^1^Nephrology and Renal Transplantation Centre, The Medical City, Baghdad, Iraq; ^2^Department of Pathology, Al-Yarmouk Teaching Hospital, Iraq

## Abstract

Allograft membranous glomerulopathy can be a recurrent or de novo disease. Both instead have different underlying immune pathophysiology and disease pattern. While the introduction of ANTI-PLAR2 and THS7A brought new insights into the management of Immune/primary MN, the treatment of de novo MN is not clear. Relapsing de novo MN in a kidney transplant was rarely reported. Here, we present a case of relapsing de novo MN without evidence of rejection and a gratifying response to rituximab.

## 1. Introduction

Membranous nephropathy (MN) is the most common cause of nephrotic syndrome in adults, with an estimated worldwide incidence of 1.2/100 000/year. [1] Immune MN is an antibody-mediated disease, with the anti-PLAR2 present in >70% of cases and other antibodies implicated like anti-thrombospondin type 1 domain-containing 7 antibody and others.

Membranous nephropathy can present in the renal transplant as a recurrence of the original disease, or de novo, and in exceptional cases as a donor-derived disease. [2] The recurrence rate varies among studies, ranging from 7% to over 44%. [3] The reported incidence of de novo MN ranges from as low as 1.5% to as high as 9%. [4]

Recurrent MN can vary in severity from a subclinical finding on biopsy to proteinuria, overt nephrotic syndrome with serious consequences including graft loss. On the contrary, de novo MN represents a separate entity with a different clinical course. [5]

With the advent of the phospholipase A2 receptor antibody (anti-PLA2R) and other antibodies, the diagnosis and treatment of primary/immune MN and post-transplant recurrence became more explicit, but it is still elusive for De novo MN. [5, 6]

Here, we report a case of relapsing de novo MN in a renal transplant with excellent response to rituximab and excellent graft function.

## 2. Case Vignette

Our patient is a 35-year-old nonsmoker male nurse who underwent a renal transplant in 2009. He presented with nephrotic syndrome at the age of 15, and his biopsy showed FSGS. There was no family history of renal disease. He received steroids and azathioprine for five years but succumbed to CKD/ESRD with hemodialysis for six months before he received his transplant. The donor was a blood group compatible spouse. Pretransplant immunology evaluation was negative with no detectable antibodies. Induction therapy included basiliximab and methylprednisolone. The graft function was excellent with no surgical or urology complications. He maintained on CSA, MMF, and oral prednisolone with an uneventful course till six years posttransplant.

In 2015, he developed proteinuria and generalized edema but with normal renal function. A renal biopsy performed and revealed membranous GN without evidence of rejection and negative C4d. All infection serology, connective tissue screen, and malignancy testing were negative. There was no evidence of de novo donor-specific antibodies (DSAs). He received two doses of rituximab 1000 mg two weeks apart. He achieved complete recovery with nil urinary protein after three months, and his renal function remained normal.

In 2019, ten years after transplantation, he relapsed with nephrotic range proteinuria. The same work-up done in 2015 was done and was negative. His serum creatinine remained at 1.2 mg/dl. A second renal allograft biopsy was again consistent with MN with no evidence of rejection and <10% IF/TA. Serum testing for anti PLA2R and AntiTHSA7 was negative as well the staining for the PLA2R and THSDA7 antigens in the biopsy. Staining for IgG subclass confirmed IgG4. He again received 2 doses of rituximab and fortunately showed the same excellent response with nil protein and normal renal function. He has no infectious complications during his disease course.

Staining for the newly discovered antigens related to MN is not available, and EM study is not available in Iraq.

During both episodes of MN, we had a comprehensive search for malignancy including abdominal and Chest CT scans, testicular and prostate ultrasound, tumor markers, and fecal occult blood. All were negative. There was no evidence of hematological malignancies with no enlarged lymph nodes, no organomegaly, and normal peripheral blood film. In addition, repeated testing for SLE and connective tissue disease was negative.

Table 1 represents laboratory and biopsy findings at one-, six-, and 10-year posttransplantation.

**Figure 1:**
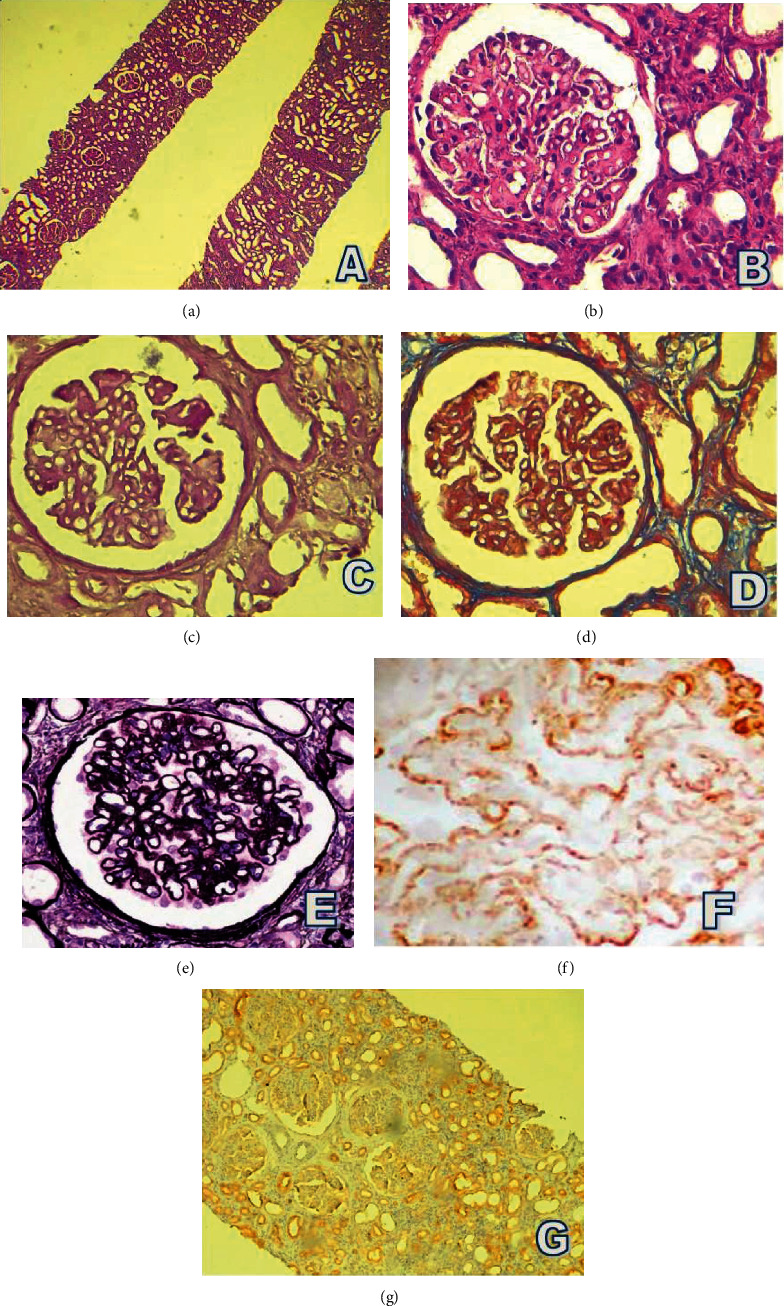
Renal allograft biopsy. (a) 40x hematoxylin and eosin (HE) stains with no apparent IF/TA, (b) 100x HE, (c) Periodic acid–Schiff (*PAS*) stain, (d) Masson trichrome stain, and (e) *Methenamine silver stain*. All (c)–(e) reveal thickened glomerular basement membrane and spikes. Immunohistochemistry (IHC) shows a granular IgG deposit (f), and it is negative for C4d, SV40, and PLA2R antigen.

Renal allograft biopsy with MN with no rejection, negative C4d, negative SV40, and negative PLA2R antigene (Figure 1).

## 3. Discussion

Here, we describe a case of de novo MN that differs from others reported in the literature. It occurs in an adult patient and recurred in an allograft that has maintained normal function without any reported evidence of rejection, nor DSA. It showed an excellent response to rituximab.

De novo MN is more prevalent in children with kidney transplants. In one report, de novo MN was present in 48 of 530 allograft biopsies in children. [4] It tends to occur many years after kidney transplantation as in this case. In two large retrospective studies, the meantime of de novo MN was 63 and 102 months, respectively. [7, 8] Despite this notion, two other reports described the early occurrence of de novo MN. [9, 10]

De novo MN may occur in patients who had end-stage kidney disease due to a different primary renal disorder. In this report, it occurs in a patient with the primary disease of FSGS. This is similar to the report of Cosyns et al. who reported de novo MN in patients with FSGS. [11] As part of podocytopathies, there may be a commonly related antigen that could be targeted by antibodies after transplantation forming immune complexes. This cannot be extrapolated as Ward et al. demonstrated circulating immune complexes in the sera of 7 patients with de novo MN, and in 5 of them, the IgG was targeted against brush border or tubular epithelial or interstitial antigens instead of podocyte antigens as seen in the primary MN. [12]

There is no well-defined pathogenic process. Several factors have been implicated that represent autoimmune, alloimmune, or peculiar host response. De novo MN may be triggered by different injuries and create an inflammatory environment, activate innate immunity, and expose cryptic antigens, different from those observed in the recurrent immune MN. This will lead to the production of circulating antibodies and in situ formation of immune complexes (IC) and the histological lesion of MN. [5, 6]

Antibody-mediated rejection and de novo DSA have been incriminated as the culprit for de novo MN with many reports, but this is not always the case. [13] Other pathologic mechanisms were implicated like hepatitis B and C, malignancy, ureteral obstruction, and recurrent glomerular diseases like IgA nephropathy and amyloidosis. [5, 6]

The occurrence of de novo MN after allogeneic hematopoietic stem cell transplantation (HSCT) may add to the understanding of the pathogenesis. It may occur as part of graft versus host disease (GVHD) or not. This indicates the formation of new immune complexes that target the podocytes. [14]

De novo MN usually does not have either circulating anti-PLAR antibodies or enhanced glomerular staining of PLA2R on kidney biopsy. Other non-PLA2R-associated MN may include THSD7A associated MN or other yet-to-be-discovered antigens. Interestingly, El Kossi described a transplant recipient who developed clinically significant de novo MN associated with typical signs of antibody-mediated damage, in the context of an HLA-DQ7 DSA. [15] Human leukocyte antigens- (HLA-) DR expression was detected on the podocytes in 8 patients with de novo MN patients. [16] Host factors may be implicated in the susceptibility to have recurrent de novo MN in more than one transplant. [4] This may be reflected in different IgG subclass in de novo MN when compared to the recurrent form, IgG1 vs. IgG4. [6] A recent report suggested that recurrent MN is driven by donor variants in HLA-D and PLA2R1loci, irrespective of PLA2R status. This will add to the complexity of the pathogenesis of MN posttransplantation. [17]

Three reports described recurrent de novo MN in the second or even a third graft. [4, 11, 18] We failed to find reports of relapsing de novo MN as in our case.

Positive staining for C4d can be a manifestation of antibody-mediated injury, but it may occur in MN as a result of complement cleavage secondary to antigen and antibody interaction. [19]

Years after the transplant, malignancy is a recognized risk. In our patient, the screen for solid or hematologic malignancies was repeatedly negative. PLA2R and THSD7A antibodies were negative. Although there is no robust evidence, PLA2R antibodies are not uncommon, and the incidence of THSD7A antibodies seems to be low in patients with cancer-associated membranous nephropathy but cannot be overlooked. [5, 20] In this report, it was unlikely to have a diagnosis of malignancy-associated MN with perfect health for ten years.

Because of such complexities, there is no consensus on treatment as it may depend on the injury mechanism. From the early reports up to the MENTOR study, rituximab showed real benefit in reducing proteinuria or inducing remission in primary MN or posttransplant recurrence. [21, 22] This may be applicable for de novo MN. [5]

De novo MN runs a variable course and variable response to therapy with graft loss in most of the cases. [5] There was one report of spontaneous remission as in the case of native primary MN. [23] This adds to the versatility and variability of de novo MN.

This report highlights the need for more understanding of the pathogenesis of MN and specifically the de novo form and should emphasize the search for hidden glomerular antigenic targets. Rituximab may be a useful tool for treating de novo MN without evident rejection or infection.

## Figures and Tables

**Table 1:** Laboratory and biopsy findings at 1, 6, and 10 years posttransplantation.

	2010 (one yr. Post-Tx)	2015 (six yrs. Post-Tx)	2019 (ten yrs. Post-Tx)
S. Cr	0.9 mg/dl	1.15 mg/dl	1.27 mg/dl
24 hrs. urinary protein	Nil	5.3 gm/24 hr	4.9 gm/24 hr
CSA c0 level	275 ng/ml	200 ng/ml	185 ng/ml
Allograft size/RI	Normal/0.5	Normal/0.6	Normal/0.6
HBV/HCV	Negative	Negative	Negative
CMV	Negative	Negative	Negative
Urine decoy cells	Negative	Negative	Negative
Biopsy			
(i) Tubulitis		Negative	Negative
(ii) Capillaritis		Negative	Negative
(iii) C4d		Negative	Negative
(iv) SV40		Negative	Negative
(v) IF/TA		<10%	<10%
(vi) PLA2R antigen		N/A	Negative
Anti-PLA2R antibody		N/A	Negative
THSDA7		N/A	Negative
ANA/anti-ds-DNA		Negative	Negative
C-ANCA/P-ANCA		Negative	Negative
C3/C4		Normal	Normal
Malignancy screen		Negative	Negative

## Abbreviations

ANA: Antinuclear antibody
Anti-ds-DNA: Antidouble strand DNA
C3: Complement 3
C4: Complement 4
CKD: Chronic Kidney Disease
CMV: Cytomegalovirus
CSA: Cyclosporin A
DSA: Donor-Specific Antibody
ESRD: End-stage Renal Disease
FSGS: Focal Segmental Glomerulosclerosis
GVHD: Graft versus Host Disease (GVHD)
HBV: Hepatitis B virus
HCV: Hepatitis C virus
HLA: Human leukocyte antigen
HSCT: Hematopoietic Stem Cell Transplantation
IC: Immune complex
IF: Interstitial Fibrosis
Ig: Immunoglobulin
MENTOR: Membranous nephropathy trial of rituximab study
MMF: Mycophenolate mofetil
MN: Membranous nephropathy
PLA2R ab: Phospholipase A2 receptor antibody
PRA: Panel reactive antibodies
RI: Resistive index
S.cr.: Serum creatinine
SV40: Simian virus40
TA: Tubular atrophy
THSD7A: Thrombospondin type 1 domain-containing 7A.
